# Topics of Public Interest

**Published:** 1908-10

**Authors:** 


### TOPICS OF PUBLIC INTEREST.

### At Last, The Oxygen Bracer.

**Rejuvenator for Work or Play—Successful Experiments on Athletes—Physicians and Surgeons Have Already Proved Its Value.**

[*New York Tribune*, September 13, 1908.]

OXYGEN for jags, for athletics and for new surgical purposes is coming to the front in a remarkable way. This gaseous element of the air, which the common people have been inhaling right along without knowing it, as M. Jourdain unconsciously talked prose, seems destined to revolutionise modern life in many directions. Uses profound and trivial are within realisation. Scientists are engaged in experiments on the effects of the gas on animals and men. Athletes are clamoring for a stimulant which has no evil after-effects and which will enable them to clip several seconds from the records. It is suggested that campaign orators who tank up on oxygen may yet be able to save the country. The fountain of youth and the elixir of life are nothing compared to the aerial element, according to some enthusiasts.

It may cause chagrin in the bosom of the average person to learn that, while he has been breathing the New York mixture that passes for air, the virtues and delights of oxygen have been monopolised by a select circle of financiers, actors, opera singers and fashionable folk. The epicure takes his oxygen highball before and after dinner. John D. Rockefeller is said to indulge moderately in this "gas of the gods," having assured himself that it is not a bad habit, like the use of drugs. The recent cavortings in the Stock Exchange might be attributed to too many oxygen cocktails consumed by brokers. Mme. Nordica warbles more divinely, it is said, when her lung cells are saturated with the Olympian element. Bernhardt would have disappointed more than one American audience if her fragile breathing apparatus had not been reinforced by the contents of several steel tanks. Sir Henry Irving was wont to tour the country in a stateroom filled with a few hundred dollars worth of the gas. The wonderful endurance of fashionable women through a winter's siege of dinners and balls is ascribed to a more or less secret indulgence in the gas that cheers but does not inebriate.

There are a myriad thrilling speculations as to the future. How soon will the subway be oxygenised? When will business men be able to carry flasks of gas in their back pockets and frequently take an enlivening draft for that tired feeling? The cost is not important, for you can get a good drink—that is, a good number of inhalations—at an oxygen parlor for $1.50, but obstacles of a mechanical nature remain. To be portable the gas must be highly compressed. A pocket flask might be as dangerous as a bomb. John G. Clifford, the president of an oxygen manufacturing company, was killed at Niagara Falls last week by the explosion of a cylinder charged with the gas to a pressure of 2,200 pounds to the square inch. His legs were torn off and hurled fifty feet away. He had been warned by conservative oxygen makers in this city that his experiments with high pressures were dangerous. The ordinary pressure used is 225 pounds to the square inch.

GIVES ATHLETES NEW VIGOR.

Other problems must be solved and questionable points settled before oxygen becomes a popular beverage, so to speak. Shall the government define a standard gas as it has defined a standard whiskey, guarantee purity and collect a revenue tax? Will the oxygen saloon be licensed and will the moralists of the latter twentieth century deplore violations of the Sunday gas law?

The first application of oxygen to athletics in this country took place at the aquatic carnival of the Chateau des Beaux-Arts, Huntington, Long Island, on August 29. The success of the application has caused many athletes to write to oxygen manufacturers asking how much gas it takes to beat the record. Chicago has enthusiastically embraced the innovation and promises oxygenated football at Marshal Field under the direction of Coach Stagg. The frightful possibilities of a flying wedge inspired with the superhuman energies of the gas do not seem to trouble the Windy City sportsmen. Perhaps they rely on the fact that oxygen medicinally applied may cure the hurts that oxygen as a stimulant has caused.

Professor Leonard Hill, of the Royal Society of London, made some athletic experiments a few weeks ago which led to the recent tests at Huntington. Dr. E. E. Smith, of No. 26 East 29th street, an exponent of experimental medicine and professor of physiology in Fordham University, after witnessing the oxygen races, made a statement to *The Tribune* representative.

"The men to whom oxygen was administered were the winners," he said. "It seems clear that its administration increases the capacity in a short struggle. It would not be scientific for me to draw indefinite conclusions from one experiment. Oxygen is not a stimulant. It merely saves the heart and lungs work by supplying to the blood the necessary oxygen which has been stored up in all the tissues, and particularly the muscle tissues of the body, by previous inhalation. The stored up supply becomes available for some time after administration. A frog muscle kept in salt solution for twenty-four hours retains its power to contract, showing that it has stored a quantity of oxygen, without which muscular action would be impossible. The indiscriminate use of the gas is not to be recommended. There are impure mixtures which are dangerous."

Dr. W. J. Geis, professor of biological chemistry in Columbia University Medical School, said:

"The experiments seem to bear out Professor Hill's work in England. Theoretically, a man's energy ought to be increased five times by breathing oxygen, since air is only one-fifth part oxygen, and that is the element which produces energy through combustion. Of course practically there cannot be so much gain. Its use calls forth reserve power and potential strength. There is not much fear of its causing overexertion. A man may walk twenty miles and lose several pounds of flesh, or row in a boat race and lose four or five pounds without injury. The danger of an oxygen habit is at present slight, if only for the reason that the gas is not obtainable in 'vest pocket' form. It is not a stimulant like morphine, nor does its effects last the whole day. It releases energy, imparts a wholesome exhilaration, makes a man feel strong and vigorous. I have no doubt its uses could be extended in these days of stress and strain. It might be useful in tiding over temporary states of weakness. Victims of gas poisoning may be resuscitated. Mining and other industries offer a field for extensive application. Our houses could be ventilated by oxygen, although at greater cost than by a regular ventilating system. The excessive use of oxygen might cause digestive trouble, because a man would have to eat unduly to make up for extra energy spent."

Edwin Fairfax Naulty, a scientific and athletic amateur who suggested and administered oxygen at the Beaux Arts races, said:

"Ray Mulvey, the swimmer, raced twice over an identical course of 100 yards. His time without oxygen was 1 minute 6 seconds; with oxygen, 58 2-5 seconds. He was not distressed at the end of his dash, while a companion showed usual fatigue. No doubt there was a psychological factor that helped Mulvey. J. Ferber, another oxygenated swimmer, made his hundred yards in 1:04 2-5, and his best previous record was 1:12. Miss Elaine Golding took oxygen before her quarter-mile race and made the distance in 8:04 2-5, swimming without distressing effort. Being unimaginative and somewhat skeptical, Miss Golding was probably not affected by suggestion. Ferber was a good subject. He said he had never swam before with such ease, and his breathing and heart action were much relieved. George Lewers took the gas and broke his under-water record, swimming about sixty yards under water in one minute 36 seconds. Mulvey took oxygen for three minutes in inhalations of forty and twenty seconds; he then rested two minutes and began his swim. Lewers had three deep inhalations and rested five minutes before his underwater swim. Ferber had about the same quantity. Miss Golding had one inhalation about an hour and a half before her swim and three inhalations immediately before it. The total results were faster time, more effective muscular effort, less distress in breathing, greater confidence, less heart action. The inhalation of oxygen before a race gives the athlete a reserve in his blood and tissues. From experiments made on myself I am certain that physical efficiency is increased. I doubt that oxygen can increase mental efficiency. I would not advise the use of it except under medical direction. The commercial product is likely to contain nitrous dioxid, which is 'laughing gas,' or chlorin. That which we used was analyzed for purity before administration. I have administered the gas to eleven athletes, with beneficial results in all cases."

USED BY WELL KNOWN PEOPLE.

Dr. Frank Northrop, of No. 6 East 45th street, informed the writer that he was the pioneer oxygen therapeutist in this country, and had administered the gas to such well known persons as John D. Rockefeller, Sir Henry Irving, Sarah Bernhardt, John Hare, Mme. Nordica, Edmond Kelley, Charles Edward Russell, Charlotte Teller and E. H. Sothern. The oil man takes a treatment whenever he has a cold or is falling down in his golf average. His friends have suggested to him that he ought to endow an oxygen dispensary, where the poor could get inhalations at a nominal charge. Sir Henry Irving spent $1,500 for gas and treatment on one tour of this country, and declared that the stuffiest dressing room and the smokiest stage hell had no terrors for him when his lungs were stored with the vitalising element. As Mephistopheles and Dante Sir Henry was pretty constantly in the infernal regions, and had especial need of oxygen. However, he was inclined to be reckless in its use, as when he flooded his stateroom with hundreds of gallons of gas while traveling. Mme. Bernhardt arrived in this country with a hemorrhage of the lungs, which was promptly stopped by the astringent effect of the gas, and she was enabled to fulfil her engagements. John Hare, suffering from heart weakness at noon before a first performance, astounded his American manager by a triumphant playing of his part at night. The physician had a tank of gas at the theatre, and kept the actor keyed up to the proper pitch, while avoiding an overdose. There was a German tenor who was to make his debut at Carnegie Hall, singing against sixteen harps, and his voice was in such a condition that he feared a signal failure. The tenor's lungs were scientifically irrigated. "I made so much noise," he reported afterward, "that they couldn't hear the sixteen harps." Singers and actors are warned, in fact, that they are in danger of shouting if they use ordinary effort after taking the gas.

Epicures who have tarried long at Sherry's or Delmonico's find it convenient to step into the oxygen parlor and get the indigestible surplus of food and drink burned out of their systems more speedily than by physical exercise and in a more refined manner than the gourmands of ancient Rome rid themselves of banquet gorges. Theoretically it would seem that these fast livers only accelerate their pace by the use of oxygen, and that they would finish their careers in about half the time required by ordinary sybarites. Fagged out business men and neurasthenic women call at the office or send for a few tanks of gas to be consumed at home. Actors, lecturers and singers are usually treated the day before a performance and are advised to eat and sleep after treatment. A natural drowsiness often follows the administration in nervous cases. Locomotor ataxia, paralysis and other diseases are said to have been cured by the treatment. Dr. Northrop does not believe in administering pure oxygen, but uses a compound of 60 per cent. oxygen to 40 per cent. nitrogen, "which is a stable and unvarying chemical combination, as fixed in composition as water itself."

According to Dr. William Seaman Bainbridge, a New York surgeon, oxygen has never had its proper place in medicine and surgery. The present revival of popular interest in the gas coincides with a revival of interest among physicians. About five years ago Dr. Bainbridge had the idea of employing oxygen in certain forms of tuberculous peritonitis. Other surgeons had used the gas to "flush out" the abdominal cavity after operations, but in Dr. Bainbridge's method the oxygen is infused into the cavity and left to be absorbed. This method had a remarkable success at the Beth Israel Hospital last July, when it was tried by Drs. Silver and Phillips. A sterile tube was inserted in an incision in the abdomen and one thousand cubic centimeters of oxygen were infused into the cavity. Then the wound was sewed up in four layers, making it airtight. Out of three cases of tuberculous peritonitis and several cases of germ peritonitis, which are usually fatal, there was only one death. The rest were cured. Presumably the gas destroyed the disease germs and stimulated the tissues toward recovery. The antiseptic power of oxygen is well known in the form of peroxide of hydrogen.

ITS USE IN MEDICINE.

The operation of "artificial pneumo-thorax" has also been tried with oxygen at Beth Israel Hospital recently. It is applied in cases of single lung tuberculosis, an incision being made in the patient's back through which oxygen is admitted, forcing the diseased lung away from the wall and giving it a rest while the healthy lung works for two. The direct benefit of the gas in tuberculosis is now being investigated. It is feared that the bacilli thrive on the oxygen almost as much as the patient.

Dr. Bainbridge conducted a series of experiments with cats last winter to determine the effects of oxygen under various conditions. He reported that it was wonderful how quickly cats and human beings responded to the gas during operations. Pulse, color and respiration were improved when oxygen was introduced in the abdominal cavity. In severe operations where the shock was very great the patient rallied almost instantly. When the lungs were washed out with oxygen after anesthesia, the patient quickly regained consciousness, felt no unusual nausea and often was able to walk from operating room to ward. Stillborn infants have been revived by the gas. It has been used subcutaneously for such diseases as carbuncle, erysipelas, sciatica and hydrocele with successful results. Asphyxiation and drug poisoning yield to the treatment. Epileptic fits have been favorably treated. Excellent results are reported from the introduction of oxygen into the intestines. Pneumonia cases have long been treated with the gas. It was administered to New York tunnel workers suffering with "the bends." Among the uses suggested by Dr. Bainbridge are inhalations before and after anesthesia, inhalations for colds and respiratory disorders, injections into joints and abscesses, and infusion into the abdominal cavity in a variety of operations. It is said the Zulus recognise the therapeutic power of oxygen by carrying their men wounded in battle to mountain tops and exposing their wounds to the air. Japanese surgeons open the abdomen in cases of tuberculous peritonitis and expose the cavity to air and sunlight for fifteen or twenty minutes at a time.

Oxygen was discovered by Scheele in Sweden in 1773, and one year later by Priestley in England. Air is a mechanical mixture of four-fifths nitrogen and one-fifth oxygen. It has not been found practicable to obtain oxygen directly from the air. The commercial method is to heat a mixture of chlorate of potash and black oxide of manganese in a retort to a temperature of 292 degrees, allowing the resultant gas to be purified by passing through a solution of caustic soda, then compressing it in steel cylinders by means of a force pump. The manganese does not yield oxygen, but merely permits decomposition of the chlorate of potash at a low temperature. One pound of chlorate will produce four and one-half cubic feet of oxygen, weighing about five and three-fifths ounces. The usual pressure of cylinders is 225 pounds to the square inch. So-called pure oxygen is about 89 per cent. with 9 per cent. of nitrogen, no chlorin and a trace of carbon dioxid. Cylinders of different sizes contain from 75 to 150 gallons of gas, retailing at $5.00 to $10.00. The Board of Health forbids the use of the inhaling apparatus, which consists of a rubber tube and a class water bottle, through which the gas passes, by more than one person. The annual production of oxygen in this country, mostly confined to New York, is said to be worth $1,000,000.

### Pasteurisation of Milk.

If farmers, dairymen and the public at large were all acquainted with the facts in regard to the relation of the milk supply to the public health, there would be little need for private, institutional or legislative measures of relief.

But unfortunately there still exists great ignorance of the danger that lurks in milk, although infected milk has become one of the most fruitful sources of disease.

When I began my work sixteen years ago, little or nothing was known of this danger. But hand in hand with my educational measures of erecting pasteurisation plants and of distributing pasteurised milk, scientists have been asking whether it was not high time to protect the public from these death-dealing germs. I had the satisfaction of seeing the death rate in New York city more than a third reduced between the year 1893, when I began my work, and 1907, when I left for Europe.

I went directly to Brussels to attend the Second International Congress des Gouttes de Lait, where physicians and scientists from the entire civilised world assembled.

I found then that in Europe even less was known of the danger of infected milk, and less attention had been paid to measures of relief, than in America. At this congress I began my European crusade by moving a resolution advising against the use of raw milk for infant feeding. This resolution was unanimously carried.

Since the adjournment of the Congress my time has been devoted to advocating pasteurisation in various parts of Europe.

For purposes of education I established a milk laboratory in Heidelberg, and supplied with pasteurised milk the Children's Hospital of the University, as well as several charitable institutions.

In Sandhausen, a village in the district of Heidelberg, I repeated an experiment which I had so successfully made in Randall's Island, in New York, many years before.

In this German village of 4,000 inhabitants the death rate among children was forty-six per hundred. I established a pasteurisation plant there. The same milk as before was used; it was simply pasteurised. Without any other change in the diet of the children, the death rate began immediately to fall.

Here is a telegram which I received from the Burgomaster of the village before I sailed:

Nathan Straus, Passenger Cedric, Queenstown:

Since February 1, 1908, there died in Sandhausen eleven children under two years of age, against twenty-five for the corresponding months in 1907 and against thirty-two average for the five preceding years. We use same milk as before, only pasteurised.

(Signed) FRANZ HAMBRECHT,
Buergermeister.

From Heidelberg I extended my activity to other cities, beginning in Karlsruhe. There I established a pasteurisation plant under the auspices of the Dowager Grand Duchess Luise of Baden. I also presented a plant to Dublin, Ireland, under the Vicereine Countess of Aberdeen. And let me say, right here, that a great deal of my European success is due to the active and moral support of these two good and noble women.

I also erected plants in Munich, under the patronage of Prinzess Arnulf of Bavaria, and in Liverpool, under the Officer of Health, Dr. E. W. Hope.

I made exhibitions which practically demonstrated the need and the benefits of pasteurisation in Frankfurt, Berlin and Vienna. Physicians, men of science, philanthropists, government and health officials, and hosts of plain good men and women who have the welfare of humanity at heart, visited these exhibitions and were unanimous in their praise of my work and my success.

The direct result of these exhibitions has been the spread of the knowledge of pasteurisation and its benefits. And the outcome will no doubt be the general introduction of pasteurisation in these cities and countries.

Baron Von Bienerth, Minister of the Interior of Austria, after visiting my exhibit in Vienna, promised to introduce the pasteurisation of milk throughout his country. To give impetus to the new work, I offered him a pasteurisation plant, which he accepted for the institution called "Des Kind." This institution was founded to commemorate the sixtieth jubilee of Emporer Franz Joseph's reign.

As the president of the Women's National Health Association of Ireland, Lady Aberdeen will exhibit in Washington at the Tuberculosis Congress, in competition for the prize offered to the organisation which has accomplished most during the last three years in the fight against tuberculosis.

Lady Aberdeen's secretary accompanied me to New York to study my method of pasteurisation at my new laboratory. She will report to Lady Aberdeen with the object of copying the work in Dublin.

The ball has been started rolling on both sides of the Atlantic, and the work is getting to dimensions which put it beyond the sphere of one man.

I owe a debt of thanks to the European press for the extraordinarily courteous treatment I received at their hands, despite my being a stranger and a foreigner. They have been constant in their support and have kept the danger of infected milk, and the remedy, pasteurisation, untiringly before the public.

With the aid of the press I hope to keep up my campaign in the future.

I earnestly hope and expect that the time is coming when no human life will be uselessly sacrificed. Typhoid fever, diphtheria and scarlet fever count their annual victims by thousands. Summer complaint counts its child victims by tens of thousands. Let us hope that this useless slaughter will be stopped. But let us not only hope.

When we know that the majority of these deaths are caused by infection through milk, let us take the only practical means of rendering milk safe.

Let us pasteurise our milk supply. Let us have for our motto: medicines and hospitals are possible cures while pasteurisation is positive prevention.

---

### THE EARLY DIAGNOSIS OF PULMONARY TUBERCULOSIS.

JOSEPH WALSH, Philadelphia, Pa., emphasises the importance of the early diagnosis of tuberculosis, and enumerates the best means of diagnosis. First we must seek the history of an exposure to contagion, in the family, in the occupation, and in the home of the patient. A history of enlarged cervical glands, pleurisy, hemoptysis, or fistula in ano is an indication of probable tuberculosis in the patient. Protracted cough, inability to take a deep breath without coughing, progressive loss of weight, gastric disturbances, and night sweats are positive indications of tuberculosis. A suggestive symptom is progressive pallor of the skin. General symptoms that are very important are evident *habitus phthisicus,* increased pulse rate, fluctuation of temperature, dyspnea, hectic flush, clubbed fingers, and bilateral dilatation of the pupils. Suggestive general symptoms are neuralgic pains, general lack of resistance, frequent chilly sensations, central or peripheral nervous disturbances, enlarged thyroid, and herpes zoster. Local symptoms that are diagnostic are change in percussion note with prolonged expiration and rales over limited areas. Very important are unilateral supraclavicular depression in inspiration, granular breathing, suppressed or muffled breathing at the apex, altered vocal fremitus or resonance, and atrophy of the scapular muscles. Suggestive are deep soreness on pressure, spinal curvature, lessened expansion on one side, lessened excursion of the diaphragm, and lessened excursion of the lung.—*Medical Record,* September 19, 1908.

